# Incidence of catastrophic healthcare expenditure and its main determinants in Mexican households caring for a person with a mental disorder

**DOI:** 10.1017/gmh.2020.29

**Published:** 2021-01-11

**Authors:** Lina Diaz-Castro, Héctor Cabello-Rangel, Carlos Pineda-Antúnez, Alejandra Pérez de León

**Affiliations:** 1 National Institute for Psychiatry Ramon de la Fuente Muniz, Direction of Epidemiological and Psychosocial Research, Mexico City, Mexico; 2 Division of Diagnostic Aids, Psychiatry Hospital Fray Bernardino Alvarez, Mexico City, Mexico; 3 Research Center on Evaluation and Surveys, National Institute of Public Health, Mexico City, Mexico

**Keywords:** Catastrophic healthcare expenditure, mental disorders, out-of-pocket expenditure

## Abstract

**Background:**

There are few studies on the impact of out-of-pocket mental health care expenditures and sociodemographic factors on the probability of Mexican households to incur catastrophic healthcare expenditures (CHE).

**Objective:**

The goal of the present study was to estimate the incidence of CHE and its main determinants among the households of persons with mental disorders (MD) in Mexico.

**Methods:**

A cross-sectional survey was conducted, including 387 households of persons with MD. The estimation of the CHE was obtained by the health expenditure distribution method. A Logistic Regression (LR) was used to identify the determinants of probability variation of CHE occurrence. Since we expected a proportion of CHE between 20% and 80%, we assume linearity in the probability function, therefore we additionally used an Ordinary Least Squares (OLS) model.

**Results:**

In our sample, the incidence of CHE was 34.8%. The two mental illnesses most frequently associated with CHE were schizophrenia and hyperactive disorder (35.5% and 32.6% of CHE cases, respectively). The regression coefficients showed that for each unit (US$53.77) increase in income, the probability of CHE was reduced by 8.6%, while for each unit increase in hospitalization or medication expenditures, the probability of CHE increased by 12.9% or 19%, respectively. For each additional household member, the probability of CHE increased by 3%, and households with a male patient had a 7% greater probability of CHE.

**Conclusion:**

Household income, household size, hospitalization and medication expenses, and sex of the patient were significant predictors of CHE for households caring for a person with MD.

## Introduction

Mental health care systems in low- and middle-income countries (LMICs), such as Mexico, are continuously in the process of reform, having an ultimate objective of reaching national health care coverage (Reyes-Morales *et al*., 2019; Ta *et al*., 2020). Guaranteeing access to the necessary medical attention for all, without financial barriers, continues to be a goal of the health care systems of all countries (Rezaei and Hajizadeh, 2019).

In Mexico, in 2017, mental disorders (MD) represented 1425 of the disability-adjusted life years (DALYs) per 100 000 inhabitants, occupying the eighth place of the global burden of diseases in the general population, while DALYs for suicide and interpersonal violence occupied third place, with 2165 DALYs per 100 000 inhabitants [Institute for Metrics and Health Evaluation (IHME), 2017*a*]. In this same year, the percent of all deaths that were related to violence in Mexico was alarmingly high: interpersonal violence (homicides) accounted for 11.6%, and suicide 1.9%, of all deaths in the general population, while these same rates for youth from 10 to 24 years old were 37.3% and 7.1%, respectively. With respect to years of life lived with disabilities, 84.1% of these corresponded to non-communicable diseases; of these, MD and substance abuse occupied first place with 19% (IHME, 2017*a*).

The magnitude of illness burden alone would be a sufficient reason for investing significantly in mental health. Unfortunately, however, the Mental Health Atlas [World Health Organization (WHO), 2015] provides data that demonstrate the scarcity of resources for attending to mental health necessities and point to inequities and inefficiencies in the distribution of these resources. In Mexico, the total expenditure on health as a percent of the gross domestic product (GDP) is 6%. While the per capita GDP is US$16 231, the per capita expenditure on health is US$664 (IHME, 2017*b*). Moreover, it has been estimated that expenditure on *mental health* is just 0.65% of the total health budget, which amounts to a per capita expenditure of just US$1.96 on mental health (World Health Organization, 2014).

On the other hand, the Mexican health system is financed by both public and private sources. Public funds attend the population that has a formal salary from the following institutions: the Mexican Institute of Social Security (IMSS), the Institute for Social Security and Services for State Workers (ISSSTE), the health services of the National Ministry of Defense (SEDENA), the Navy (SEMAR), and Mexican Petroleum (PEMEX). Public funds also attend the population that does not have a formal salary through the Secretary of Health (Secretaría de Salud). Health institutions for the insured population are funded by contributions from the corresponding employer, federal government, and the worker. The health institutions for the uninsured are sustained by funds from the State and Federal Governments, and to a much lesser extent by contributions from the beneficiaries. Private health care services are financed by insurance premiums and out-of-pocket (OOP) expenditure at the moment at which health care is received (Arredondo *et al*., 2018).

According to health indicators of the World Bank, 49.4% and 46.5% of health care expenditures in Mexico are, respectively, covered by public and private resources, the latter comprising household OOP expenditures (The World Bank, 2013). Moreover, household OOP health care expenses have steadily increased as a proportion of total health care expenditures, reaching 41.3% in 2017, compared to the decreasing trend that was seen across 2003–2013 (from 55% to 39.3%; Secretaría de Salud, 2019).

There are few empirical studies on the impact of the financial burden of such OOP contributions for Mexican households, or on the sociodemographic characteristics of the affected households or associated health expenditure (Knaul *et al*., 2018).

The objective of the present study was to estimate the incidence of catastrophic healthcare expenditure (CHE) and the determinants associated with incurring such expenses, in households that include a person with MD. This study extends our knowledge and awareness of the need to protect the financial situation of people with MD.

## Methods

An exploratory cross-sectional study was carried out in two national reference psychiatric hospitals in Mexico: The Fray Bernardino Álvarez Psychiatric Hospital or Hospital Psiquiátrico Fray Bernardino Álvarez (HPFBA) and the Dr Juan N. Navarro Children's Psychiatric Hospital or Hospital Psiquiátrico Infantil Dr Juan N. Navarro (HPIJNN), during January to October of 2018. The protocol was approved by the Ethics and Investigation Committees of both hospitals (Registry 782 and II3/02/0917, respectively). All participants agreed to participate in the study and signed an informed consent form.

### Sample population and data collection

#### Setting

The HPFBA is the largest psychiatric hospital in Mexico, with 300 hospital beds and 22 outpatient consultation rooms, a day hospital, an intensive care unit, laboratory, imaging area, and emergency room. The HPFBA attends to adult psychiatric patients (age 18 or more) that are not affiliated with social security (i.e. that do not receive a formal salary). The HPIJNN is the psychiatric hospital in Mexico that provides attention to children and adolescents under 18 years old, and not affiliated with social security. The HPIJNN has 95 hospital beds for acutely ill patients, 20 outpatient consultation rooms, laboratory, imaging area, and emergency room.

#### Sample population

A probabilistic sample of 387 face-to-face interviews, using simple random sampling. The unit of analysis was the household of an outpatient with a diagnosis of an MD and that was receiving medical attention from one of the selected hospitals. Information on the patient's diagnosis was confirmed from his/her clinical file. Diagnoses were determined by a psychiatrist, and based on the criteria of the International Classification of Diseases [World Health Organization (WHO), 2016]. Households in which the patient did not have a diagnosis, or in which the patient's primary caregiver was not available for interviewing, were excluded from the study. Also excluded were cases in which the caregiver did not record household expenses and/or health care expenses.

#### Questionnaire

The questionnaire that was administered was designed based on questions contained within the National Survey of Household Income and Expenses 2018, Mexico (INEGI, 2018). This questionnaire was administered to the patient's primary caregiver, that is, the principal person that provides treatment and care to the patient. Incomes and expenses were converted to US dollars, according to the exchange rate at the time of the study (US$1 = 20.48 Mexican peso; 15 June 2018).

### Study variables

#### Sociodemographic variables

The variables related to the patient as well as to the caregiver were: sex, age, years of education, size of the households (number of individuals living in the household), marital status [single = not having a stable relationship with a partner, being a widow(er), or divorced; married = having a stable relationship, married or other civil union], and occupation. In the case of occupation, the classification described by the WHO was applied: paid work (formal employment), self-employed, student, housekeeper, unemployed (health reasons), unemployed (other reasons), retired, and other (Vazquez-Barquero *et al*., 2000).

#### Household income

This refers to income from salary, retirement benefits, remittances, and support from government or private programs for obtaining medicines.

#### Total household expenditures (THE*_h_*)

THE*_h_* is defined as the total expenditures of the households, including food, clothes, transportation, services, etc.

#### OOP health care expenditures

OOP health care expenditures is defined as the total of all expenditures made by the households for psychiatric attention, including expenses for consultations, hospitalizations, medicines, diagnostic tests, and transportation. The OOP health care expenditures is a continuous variable bounded by zero that can have any positive value.

Household expenditures and income obtained during the previous month was multiplied by 12 in order to obtain annual measures.

#### Poverty line

The poverty line was calculated based on the THE*_h_*, taking into account the adjusted number of individuals per household, as described by Xu (2005).

#### Subsistence expenditure (SE)

SE*_h_* is defined as the minimum expenditure, adjusted for household income, that is necessary for the households to remain above the poverty line (Xu, 2005).

#### Payment capacity (PC)

The PC for a given household (PC*_h_*) was then calculated as the income above the poverty line: PC*_h_* = THE*_h_*–SE*_h_* (Xu, 2005). In most cases, the PC*_h_* was a positive value, since the expenditures on food are contemplated within the THE*_h_*. However, in some of the very poor households, the calculated PC*_h_* had a negative value. In these cases, the poverty line was substituted by the total household expenditure on food (Xu, 2005).

#### Catastrophic healthcare expenditures (CHE)

CHE*_h_* is defined as the OOP health care expenditures that exceed a specific threshold during the year. The threshold most accepted and recommended by the WHO is a certain percentage of the PC*_h_* (Hailemichael *et al*., 2019). In the case of Mexico, a threshold of 30% of the PC*_h_* has been applied (Sesma-Vázquez *et al*., 2005; Knaul *et al*., 2018). Thus, a household *h* incurs catastrophic healthcare expenditures (CHE*_h_*) if health care expenditures amount to more than 30% of its capacity to pay (PC*_h_*). For the analysis, CHE*_h_* was assigned a value of 1 or 0: CHE*_h_* = 1, if (OOP health care expenditures*_h_*/PC*_h_*) was ≥0.30; and CHE*_h_* = 0, if (OOP health care expenditures*_h_*/PC*_h_*) was <0.30. Threshold levels that are considered for calculating the CHE can vary between countries, for this reason, a sensitivity analysis was done using several different thresholds (10, 20, 30 and 40%) of the PC*_h_*, in order to compare these to our selected threshold of 30%.

#### Incidence of households having catastrophic healthcare expenses

This was defined by the following equation: 
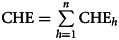
 (Knaul *et al*., 2018). The CHE*_h_* was a categorical variable with values of yes or no (Lee *et al*., 2019).

### Statistical analysis

For all analyses, STATA version 14 software was used. For descriptive analyse, frequencies, percentages, or measures of central tendency were calculated, depending on the nature of the variable. The estimation of CHE was obtained by the method of health expenditure distribution (Xu, 2005). A Logistic Regression (LR) model was used to identify the determinants of probability variation of CHE occurrence. Since we expected a proportion of CHE between 20% and 80%, we assume linearity in the probability function, therefore we additionally used an Ordinary Least Squares (OLS) model (Fitzmaurice *et al*., 2004). The stepwise entry-removal of the various explanatory variables allowed identifying those that had a statistically significant influence on the probability of determining CHE.

## Results

### Descriptive analyses

Table 1 shows the sociodemographic characteristics of the study sample (387 households) that were significantly associated with CHE. It is important to mention that the majority of patients in the present study were men (74%), while the majority of their caregivers were women (86%).

**Table 1:** Sample characteristics of households with mental disorders associated with CHE

	Catastrophic health expenditure					
	No		Yes			
Categorical measures	Frequency	%	Frequency	%	% Total	χ^2^
Sex (patients)						7.4***
Female	77	76.2	24	23.8	26.1	
Male	175	61.2	111	38.8	73.9	
Sex (caregivers)						0.003
Female	217	65.2	116	34.8	86.0	
Male	35	64.8	19	35.2	14.0	
Diagnostic						32.7***
Anxiety disorder	5	1.3	0	0.0	1.3	
Unspecified mental disorder	3	0.8	2	0.5	1.3	
Personality disorder	6	1.6	5	1.3	2.8	
Depressive disorder	7	1.8	8	2.1	3.9	
Another psychotic disorder	17	4.4	8	2.1	6.5	
Substance use disorder	58	15.0	20	5.2	20.2	
Schizophrenia	35	9.0	48	12.4	21.4	
Hyperkinetic disorder	121	31.3	44	11.4	42.6	
Household size						5.7*
1–3 members	116	30	77	19.9	49.9	
4–6 members	128	33.1	57	14.7	47.8	
>6 members	8	2.1	1	0.3	2.3	
Employment status						26.4***
Formal employment	6	1.6	6	1.6	3.1	
Keeping house	15	3.9	3	0.8	4.7	
Self employed	17	4.4	6	1.6	5.9	
Unemployed (other reasons)	15	3.9	8	2.1	5.9	
Unemployed (health reasons)	37	9.6	48	12.4	22.0	
Student	162	41.9	64	16.5	58.4	
Continuous measures	Mean	s.d.	Mean	s.d.		*t* test
Age of patients (years)	18.5	14.2	23	14.9		−3.0***
Schooling years (patients)	5.3	4.0	6.8	4.4		−3.3***
Age of caregivers (years)	43.2	11.7	47.8	12.7		−3.6***
Schooling years (caregivers)	5.3	4.0	6.8	4.4		−3.4***
Households income	$365	$239	$275	$148		4.0***
Households payment capacity	$227	$207	$274	$117		3.8***
Out-of-pocket						
Transportation	$11.6	$5.7	$8.8	$5.4		0.26
Food	$2.9	$2.5	$3.2	$3.1		−2.0*
Outpatient services	$1.4	$1.3	$2.8	$2.0		−2.6**
Hospitalization	$27.4	$6.5	$79.2	$59.7		−9.7***
Medicines	$26.5	$25.0	$46.5	$46.0		−5.4***
Lost days of employment	5.3	2.4	9.4	6.3		−3.7***

$ = USD; US$1 = 20.48 MXN (Mexican peso, 15 June 2018).

***p < 0.001, **p < 0.01, *p < 0.05.

The mean age of the primary caregiver was 45 (s.d. = 12), with an average of 11 years of formal education (s.d. = 3.7), and the marital status of the majority of them was single (92.8%). In the majority of households, the caregiver was the mother of the patient (71%, *n* = 273), followed by one of the siblings (8%, *n* = 29), the grandmother (6%, *n* = 23), while in the rest of the cases, it was the patient's partner, aunt, daughter/son, or tutor.

The prevalence of CHE in this study sample was 34.8%. The households that incurred CHE were those that provided pharmacological treatments to patients with a schizophrenia diagnosis (35.5% of the total households with CHE) and hyperactive disorder (32.6%). By contrast, no households that attended to a patient with a diagnosis of anxiety disorder incurred CHE (χ^2^ = 32.7; *p* = 0.000).

Households with male patients were more frequently associated with CHE, compared to those having a female patient (χ^2^ = 7.4; *p* < 0.001). In fact, the only study variable that was not significantly associated with CHE was the sex of the caregiver (see Table 1).

**Fig. 1.**
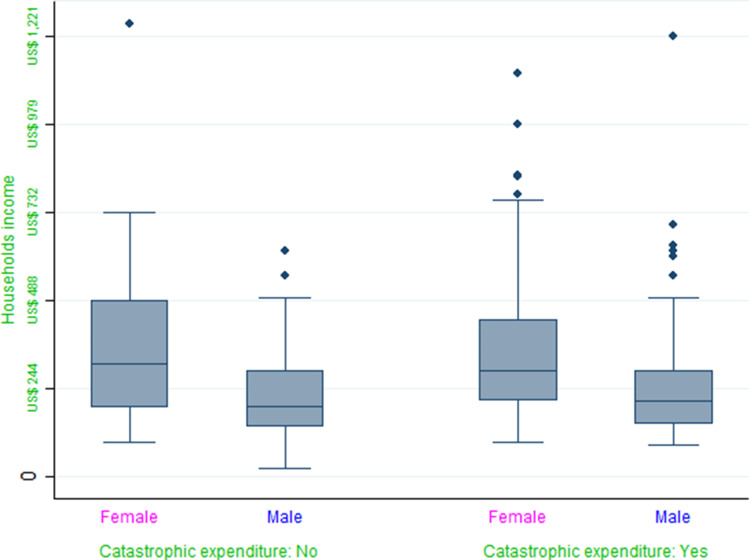
Catastrophic expenditure by patient sex and household income.

With respect to monthly income, households had an average monthly income of US$338.3 (s.d. = 219.2), and mean incomes were significantly different between households that did or did not incur CHE. It is notable that lower mean incomes were associated both with an increased probability of CHE, as well as with households in which the patient was male (Fig. 1).

With respect to the level of education, male patients of households that incurred CHE had an average of 4 fewer years of formal education.

Results showed significant differences in average household income and average household expenditures associated with CHE: in general, households that incurred CHE spent a significantly greater proportion of their income on medical attention, those being direct expenses of medical attention as well as expenses for accessing this attention (see Table 1).

### Regression analyses

Results of LR and OLS regression are shown in Tables 2 and 3. For a direct comparison between both models, marginal changes were estimated in the LR model. In total, 387 households were analyzed, but three cases were discarded due to incomplete data of health care expenses. Regression coefficients of LR and OLS models were similar in magnitude and all showed the same direction of effect on probability. Therefore, the OLS model was appropriate for modeling the probability to incur CHE.

**Table 2.** Logistic Regression models to identify determinants of variation in the probability of CHE

	Logistic	Logistic
Variables	Catastrophic expenditure 30%	Catastrophic expenditure 40%
Patient sex (male)	0.028	0.070*
	(−0.045 to 0.102)	(−0.007 to −0.147)
Schooling years (patients)	−0.007	−0.002
	(−0.018 to 0.005)	(−0.013 to 0.010)
Schooling years (caregivers)	−0.000	−0.006
	(−0.010 to 0.009)	(−0.016 to 0.004)
Households size	0.032**	0.029**
	(0.006–0.059)	(0.002–0.055)
Households income	−0.086***	−0.090***
	(−0.100 to −0.073)	(−0.102 to −0.077)
Employment status	0.143	0.066
	(−0.093 to 0.379)	(−0.152 to 0.284)
Self-employment	−0.165	−0.147
	(−0.432 to 0.102)	(−0.412 to 0.119)
Work days lost	−0.006	0.010
	(−0.023 to 0.011)	(−0.005 to 0.025)
Hospitalization expenditure	0.393***	0.304***
	(0.311–0.474)	(0.244–0.365)
Medicines expenditure	0.426***	0.348***
	(0.371–0.481)	(0.289–0.408)
Observations	384	384
*R*^2^	0.540	0.545

*Note*: Given the high proportion of catastrophic expenditures observed when the thresholds of 10% and 20% were used, the Logistic models did not achieve the convergence.

CI 95% in parentheses.

***p < 0.01, **p < 0.05, *p < 0.1.

**Table 3.** OLS regression models to identify determinants of variation in the probability of CHE

	OLS	OLS	OLS	OLS
Variables	Catastrophic expenditure 10%	Catastrophic expenditure 20%	Catastrophic expenditure 30%	Catastrophic expenditure 40%
Patient sex (male)	0.035	0.082*	0.086*	0.119**
	(−0.052 to 0.121)	(−0.014 to −0.179)	(−0.010 to −0.182)	(0.026–0.212)
Schooling years (patients)	−0.002	0.008	0.007	0.011*
	(−0.013 to 0.009)	(−0.004 to 0.020)	(−0.005 to 0.019)	(−0.001 to −0.023)
Schooling years (caregivers)	−0.008	−0.007	−0.006	−0.011*
	(−0.019 to 0.002)	(−0.018 to 0.005)	(−0.018 to 0.005)	(−0.022 to −0.000)
Household size	0.022	0.013	0.018	0.016
	(−0.009 to 0.054)	(−0.022 to 0.048)	(−0.017 to 0.053)	(−0.018 to 0.051)
Households income	−0.032***	−0.046***	−0.045***	−0.045***
	(−0.041 to 0.022)	(−0.057 to 0.036)	(−0.055 to 0.034)	(−0.056 to 0.035)
Employment status	−0.023	0.001	0.161	0.126
	(−0.247 to 0.201)	(−0.250 to 0.252)	(−0.089 to 0.411)	(−0.116 to 0.368)
Self-employment	−0.373***	−0.264*	−0.404***	−0.363**
	(−0.635 to 0.112)	(−0.557 to 0.030)	(−0.696 to 0.112)	(−0.646 to 0.081)
Work days lost	0.005	0.004	0.005	0.008**
	(−0.001 to 0.011)	(−0.002 to 0.011)	(−0.002 to 0.012)	(0.001–0.014)
Hospitalization expenditure	0.055***	0.094***	0.129***	0.122***
	(0.015–0.096)	(0.049–0.139)	(0.084–0.174)	(0.079–0.166)
Medicines expenditure	0.190***	0.213***	0.191***	0.164***
	(0.134–0.247)	(0.149–0.276)	(0.128–0.254)	(0.103–0.224)
Constant	0.811***	0.615***	0.435***	0.392***
	(0.629–0.992)	(0.411–0.818)	(0.232–0.638)	(0.196–0.588)
Observations	384	384	384	384
*R*^2^	0.273	0.322	0.348	0.364

CI 95% in parentheses.

***p < 0.01, **p < 0.05, *p < 0.1.

The logistic model considering a threshold of 30% of the PC*_h_* was significantly accurate (χ^2^ = 289, gl = 10, *p* ≤ 0.001). Considering the pseudo *R*^2^, the model explained 54% of the variance in CHE.

Household size, income, hospitalization expenditure, and the cost of medicines were significant predictors of CHE (Table 2). Regression coefficients showed that for each increase in the household income of US$53.77 (1000 Mexican Pesos), the probability of CHE was reduced by 8.6%. By contrast, for each increase of US$53.77 in additional hospitalization costs, the probability of CHE increased by 12.9%, and for each increase of $53.77 in additional costs of medicine, the probability of CHE increased by 19% (Table 3).

Households in which the patient was male had a 7% greater probability for CHE compared to those with a female patient; however, this result was statistically significant only in the model that considered a threshold of 40% of the PC (Table 2).

The size of the household showed a statistically significant effect on CHE: the probability for CHE increased by 3% for each additional household member (Table 2).

Based on the results of the OLS model considering a threshold of 30% of the PC, we observed a general consistency between the OLS and logistic model. However, the OLS model identified additional statistically significant factors, including missed days of work due to accompanying while the patient receives medical attention. Thus, for each day of work missed, the probability of CHE increased by 5%. On the other hand, households in which the patient was able to maintain his/her employment showed a 40% lower probability of CHE (Table 3).

Finally, considering a threshold of 30% of the PC*_h_*, the regression coefficients in both models were similar in magnitude and in the direction of the effect on probability, and were found to be adequate for predicting CHC in the household sample of the present study (Tables 2 and 3).

## Discussion

Financial protection of households against CHE is a principal goal of health care systems (Rezaei *et al*., 2019). Nevertheless, the results of the present study show that all studied households had OOP health care expenditures, and a large proportion of households (35%) incurred CHE, a proportion similar to that reported in another recent study (Hailemichael *et al*., 2019).

The present results should be evaluated within the perspective that, although it is difficult to achieve financial protection from health care expenditures due to their unpredictable and sometimes catastrophic nature (Knaul *et al*., 2005), the households in the present study did have the possibility of access to free medical attention that would have included medicines, hospitalization and diagnostic studies. Despite this possibility, more than a quarter of households that sought medical attention at the psychiatric hospitals for nevertheless incurred CHE. Therefore, although the possibility for access to free medical attention existed for our study population, in practice the patients were unable to take advantage of this possibility. In this light, a major factor for incurring CHE in the present study population was the lack of effective strategies for putting into practice existing programs and policies that would have offered financial protection (Berenzon-Gorn *et al*., 2018).

In Mexico and in other LMICs, it has been reported that households with a person receiving medical attention for an MD incur more CHE compared to those households that receive general medical attention (Grogger *et al*., 2015). However, several studies have shown different results: for example, in Mexico, lower CHE has been reported for diabetes treatment and control (Sosa-rubí and López-ridaura, 2009), and for cataract surgery (Navarrete-lópez *et al*., 2013). Nevertheless, in a study of a population of elderly adults with chronic illnesses such as diabetes and cancer, no positive effect of the program ‘Popular Insurance’ (‘*Seguro Popular*’) was observed on CHE (Rivera-Hernández *et al*., 2019).

In the case of persons with MD, aside from the availability of strategies to prevent CHE, mechanisms of access for protecting households from health-related financial risks are not widely available, as has been reported in the literature (Woldemichael *et al*., 2019). Moreover, there is a disparity between medical attention provided to the population for physical health and attention provided for mental health (Lavie-Ajayi *et al*., 2018), due in part to the extent of poverty, the segmentation and fragmentation of the health system, and the lack or unequal distribution of financial, physical, and human resources in the health system (Laurell, 2013).

The present results showed that households having a person with an MD had a mean lower income level compared to the general population (US$338 *v.* US$807; Naciones Unidas México, 2020). Similarly, the high rates of unemployment or informal employment in these households that were observed in the present study have been reported by others (Banerjee *et al*., 2017; Mufson and Rynn, 2019). These factors only amplify the situation of social inequality and inequity in households caring for a person with an MD.

In this respect, even though it has been reported that health insurance coverage protects against expenditures that are considered catastrophic (Tirgil *et al*., 2019), it is not sufficient: according to the present results and other reports in the literature, protection rather depends on the social context of the health system and other tangible barriers to access (Kisely *et al*., 2009; Gal *et al*., 2017; Erlangga *et al*., 2019; Kim *et al*., 2019; Ta *et al*., 2020). In this sense, strategies for healthcare for MD is centered on the capacitation of human resources, in strengthening the health system, as well as in the direct provision of medical attention to mental health (Hanlon *et al*., 2018), with the understanding that expenses for hospitalization and medicine are principal causes of CHE, as demonstrated in the present study sample.

In the case of medical attention for schizophrenia, the MD with the highest incidence in the present study (12.4%, Table 1), households incurred expenses for hospital and outpatient services in addition to expenses for lifelong medication. Our results indicate that the probability of CHE in these cases would have been decreased by 13% and 19%, if these households had access to financial protection for hospital costs or for medication costs, respectively. By means of comparison, in Peru, expenses for medicines represented 44% of OOP expenditure for families with social security, compared to 62% for families that were not affiliated with this program (Petrera Pavone and Jiménez Sánchez, 2018), indicating the necessity to incorporate strategies for increasing both availability as well as accessibility of financial protection for households caring for a person with an MD.

It has been documented that households of persons with severe MD such as schizophrenia adopt strategies for facing financial challenges, such as reducing hospital visits, reducing food consumption, or even taking children out of school (Hailemichael *et al*., 2019), which in the long term have a greater negative impact. This finding gives more support to our results, in the sense that these households face adverse social conditions such as lower formal education, lower probability to have formal and well-paid employment, and consequently have lower income. In this context, it is notable that in the present study, we found that household income level was a major determinant of the probability of CHC, and similar results have been reported in other studies (Zuvekas and Selden, 2010; Rezaei and Hajizadeh, 2019; Tirgil *et al*., 2019; Woldemichael *et al*., 2019).

The phenomenon of disparities in medical attention to mental health is a complex problem in Mexico as well as in other LMICs (Shadmi, 2018). In general, households caring for a person with an MD have more difficulties with respect to access to medical attention (Thornicroft, 2018) along with lower affiliation with social security and other types of health insurance. As a result, such households come to incur enormous costs of medical attention (Cotgrove, 2018; MacDonald *et al*., 2018; Xu *et al*., 2019) and a greater incidence of CHE.

The problem lies in that households caring for a person with an MD require more information about the availability of health services and how to gain access to them (Iskra *et al*., 2018). Likewise, if persons with MD are to have access to medical attention and financial equity, a comprehensive approach will be required in order to identify and address the social determinants that act to perpetuate unfavorable life conditions and incidence of CHE (Callander *et al*., 2019).

To this end, the recent health system reform in Mexico, as part of the recognition that the right to health is essential and irrevocable, makes explicit the central role of the state for promoting and adopting laws as well as for providing monetary support in order to guarantee access to medical attention. It has consistently been true that more than 50% of the Mexican population lack access to social security (Reyes-Morales *et al*., 2019). In order to address this issue, the System of Social Protection of Health (*Sistema de Protección Social en Salud*; *SPSS*), and its program ‘*Popular Insurance*’ (‘*Seguro Popular*’) was abolished in order to put in place the Institute of Health for Well-Being (*Instituto de Salud para el Bienestar*; *INSABI*). If indeed the strength of the INSABI is the proposal to provide free health care service and medicines, which would have a tremendous positive impact on households through eliminating CHE, it is necessary to reflect about mechanisms and flow of the finances of INSABI, as well as about sustainability. Along these lines, we suggest that for the initiative to be viable, it should incorporate experts in this area that provide information based on the best evidence available, for the sake of having scientific and empirical support for its objectives and substantive phenomena of health systems.

## Limitations

There are several limitations to the present study. First, the study was carried out in two psychiatric hospitals in Mexico City, in a population without social security; for that reason, the interpretations of these results are limited to this population group. The sample was not a representative sample of the population of persons with acute or exacerbated MD, or of the households of this population. Likewise, the social conditions of vulnerability identified in the present study might not be representative of the population of households caring for a person with an MD, as a whole. The specific social condition characteristic of the present study sample may have been a factor in determining the overall elevated prevalence of CHE that we observed; for this reason, additional studies are necessary that would incorporate a household population that would be more representative of the population as a whole.
